# Supplementary material for: Uncovering the molecular mechanisms of russet skin formation in Niagara grapevine (Vitis vinifera × Vitis labrusca)
Source: Sci Rep. 2024 Mar 19;14:6600. doi: 10.1038/s41598-024-55745-8 (PMC10950848; doi:10.1038/s41598-024-55745-8)
Supplement: Supplementary file 1 — Supplementary Figures. [file 41598_2024_55745_MOESM1_ESM.pdf]

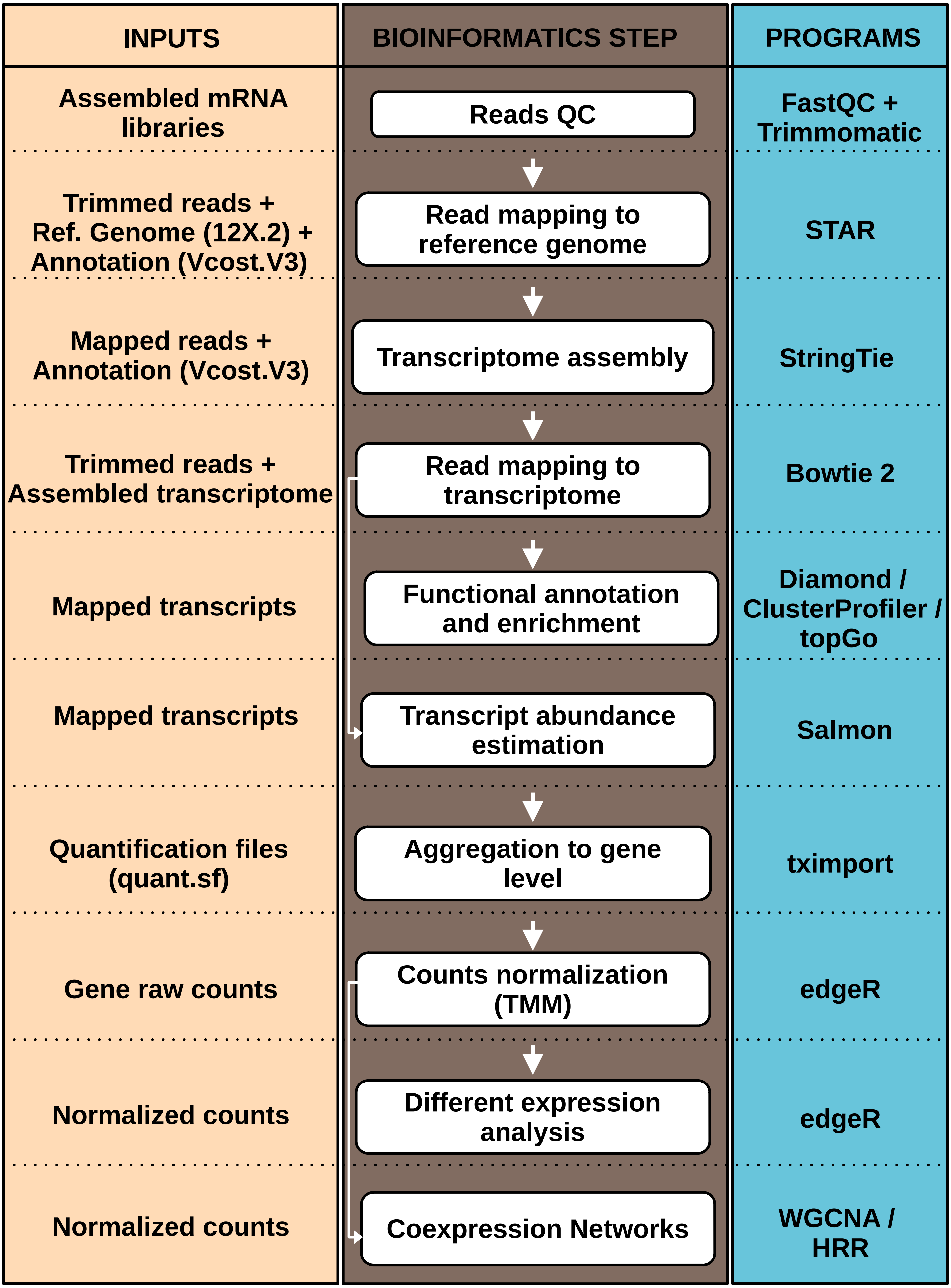

Supplementary Figure S1: Overview of the transcriptome analysis pipeline. StringTie was selected for its capability to assemble both *de novo* transcripts and genome-based ones, providing a comprehensive approach to address potential genomic variations in studied genotypes compared to the reference.

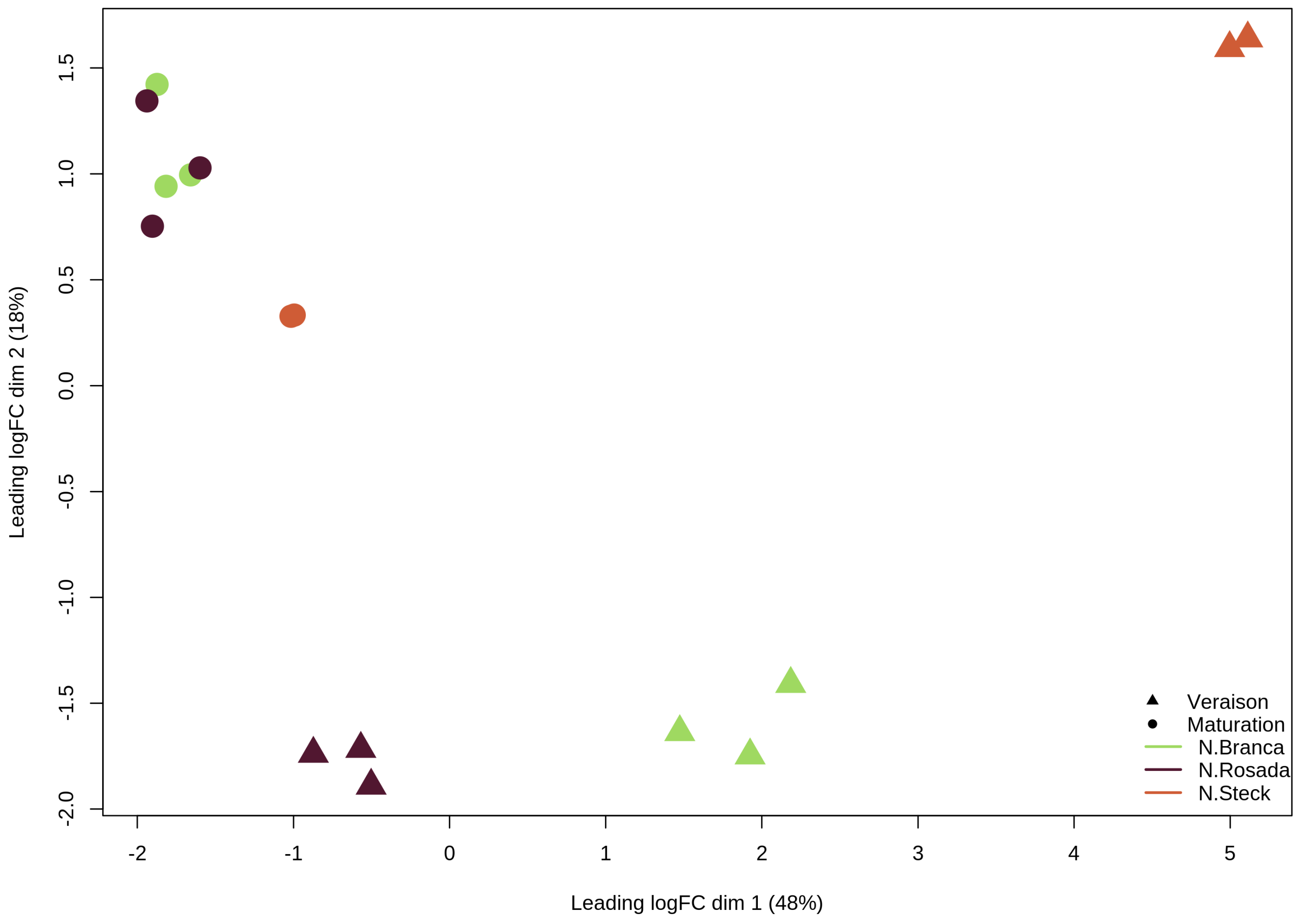

Supplementary Figure S2: Principal Components Analysis (PCA) of gene counts for sample triplicates, emphasizing the homogeneity of biological replicates.

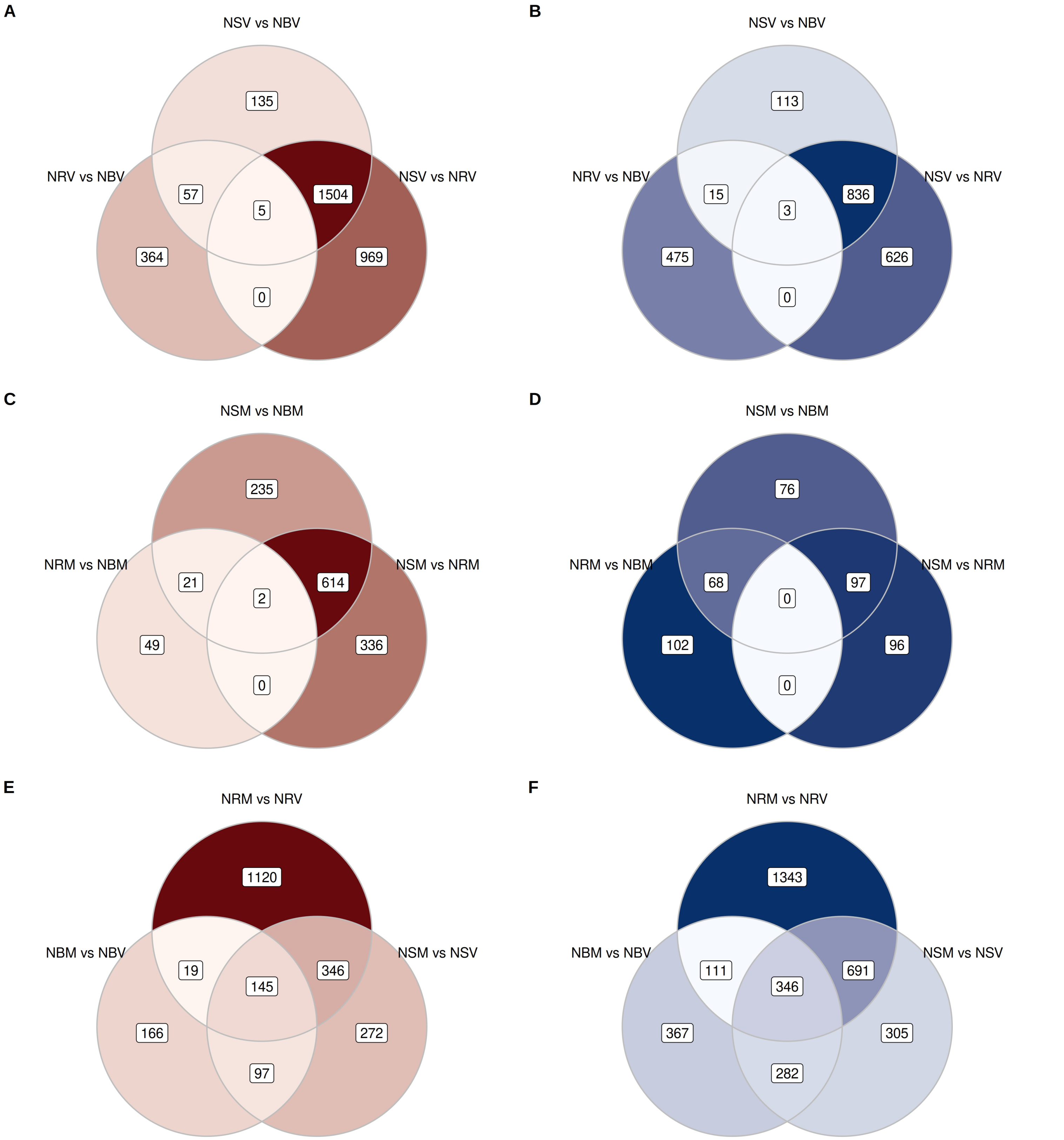

Suppplementary Figure S3: Venn Diagrams illustrating the intersection of overall differentially expressed genes across contrasts based on their logFoldChange. Positive regulation intersections during veraison (A), maturation (C), and within genotypes throughout ripening (E). Negative regulation intersections during veraison (B), maturation (D), and within genotypes throughout ripening (F).

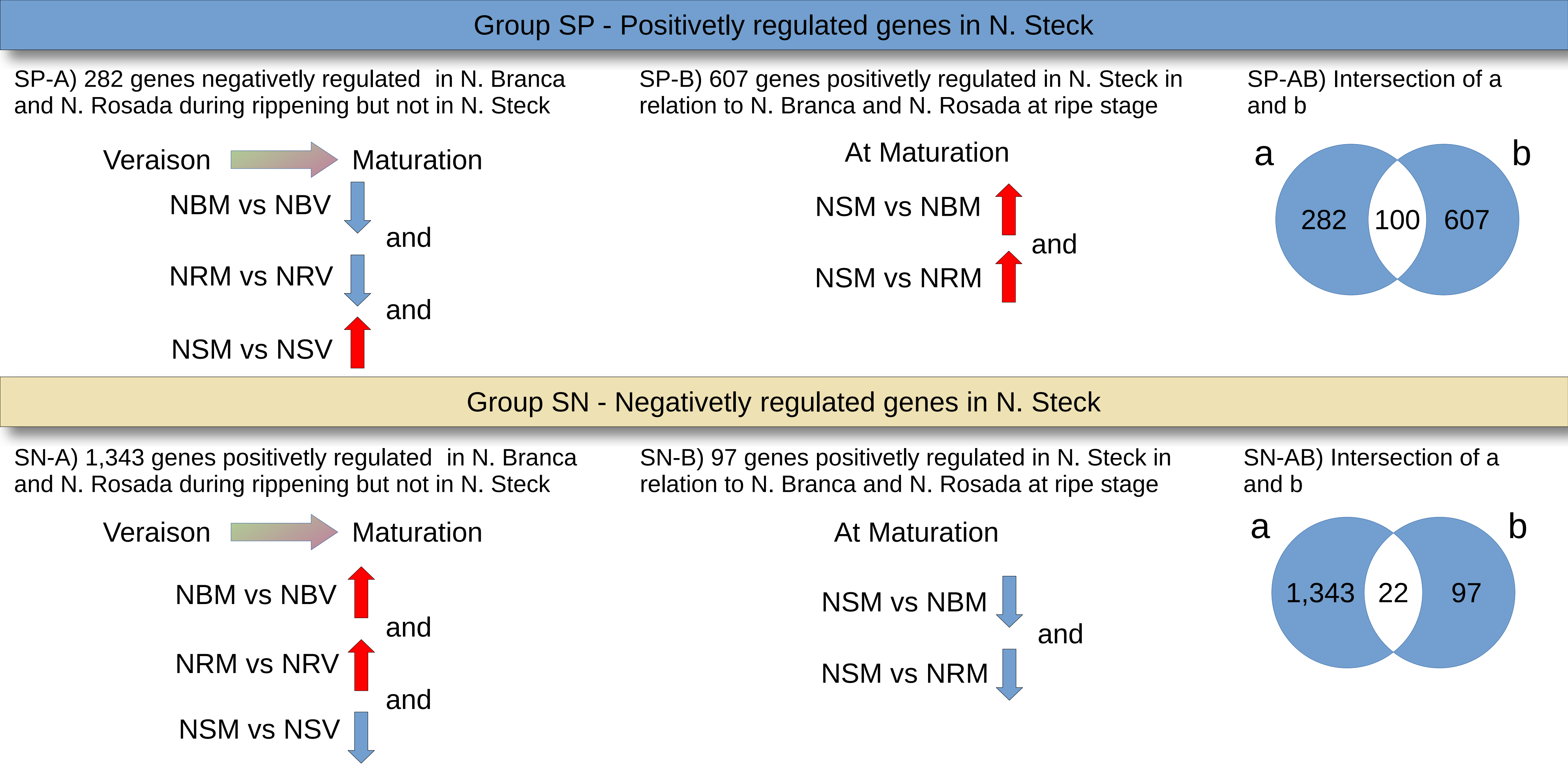

Supplementary Figure S4: Schematic outlining the methodology to isolate genes specifically associated with the N. Steck genotype. This approach aims to exclude genes responsible for individual differences between N. Steck and other genotypes, focusing on highlighting genes consistently differentially expressed in N. Steck regardless of the comparison.

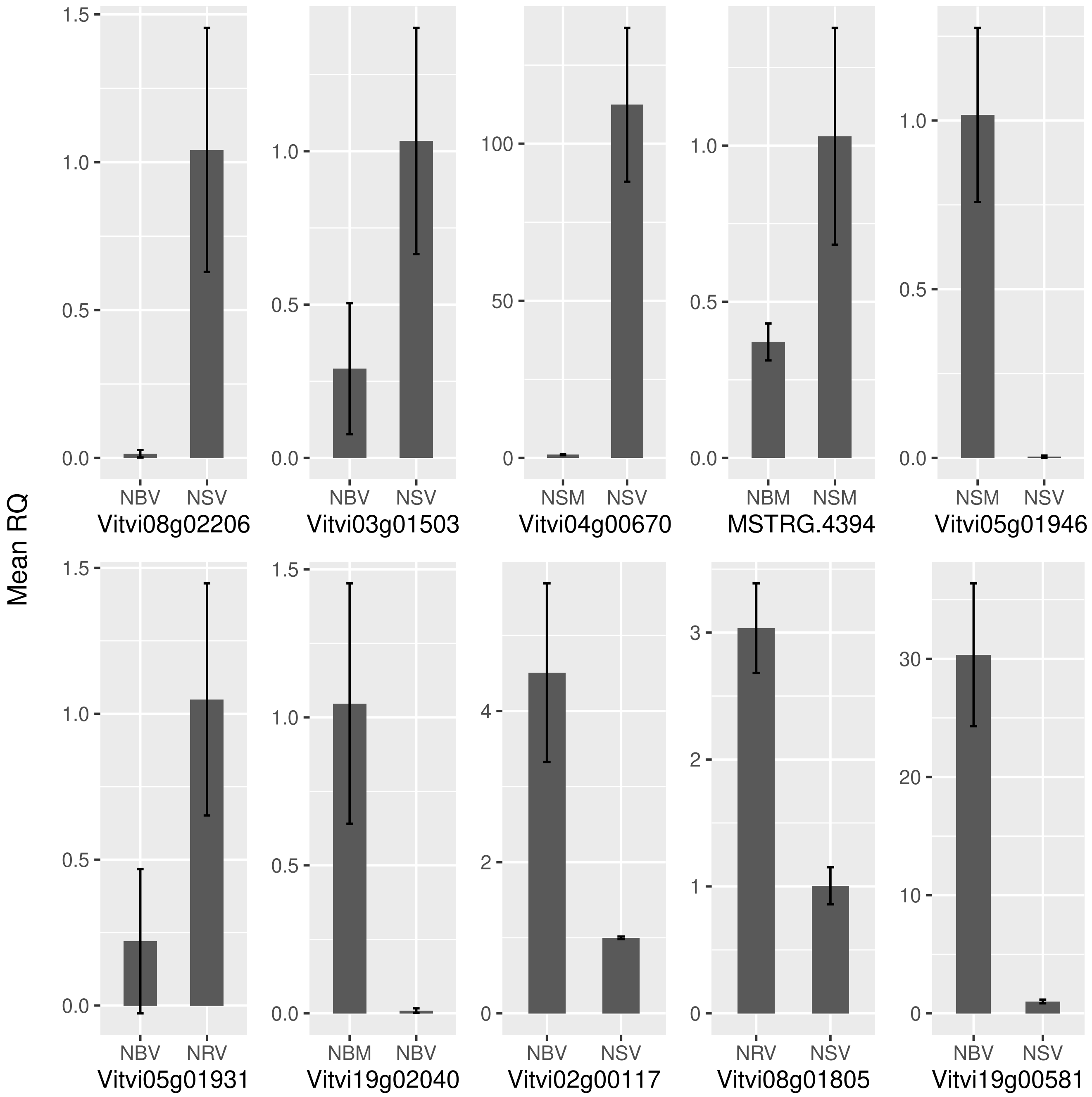

Supplementary Figure S5: Validation of the results from differential expression analysis. RT-qPCR technique was conducted using ten differentially expressed genes selected at random. Their corresponding cycle quantification (Cq) values were utilized to apply the ΔΔCq method, thereby determining the Relative Quantification (RQ) values. Statistical significance of the analysis was evaluated using the Student's t-test.

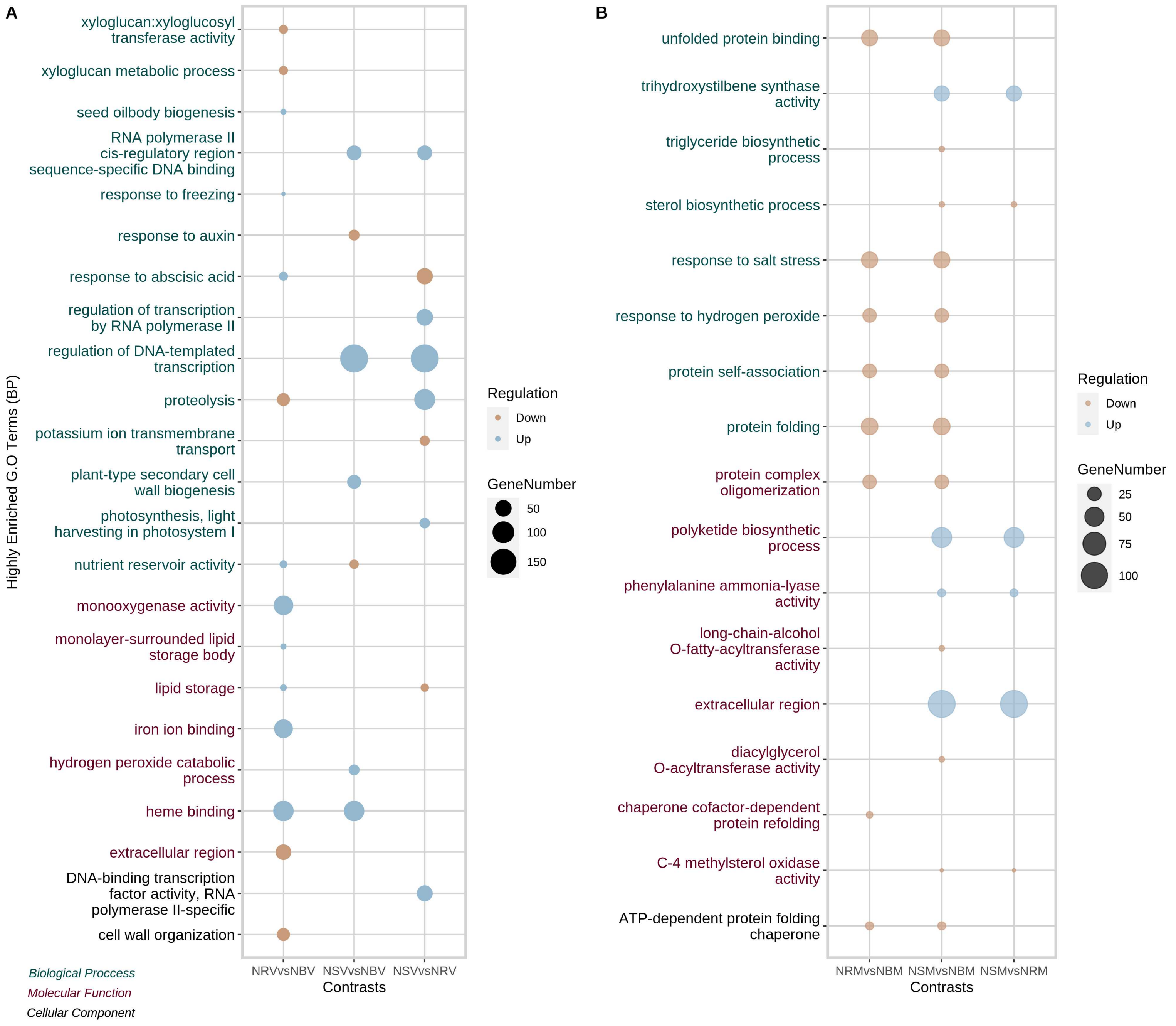

Supplementary Figure S6: Scattergrams of enriched GO terms in expression contrasts between genotypes at both berry development stages. A) Veraison. B) Maturation.

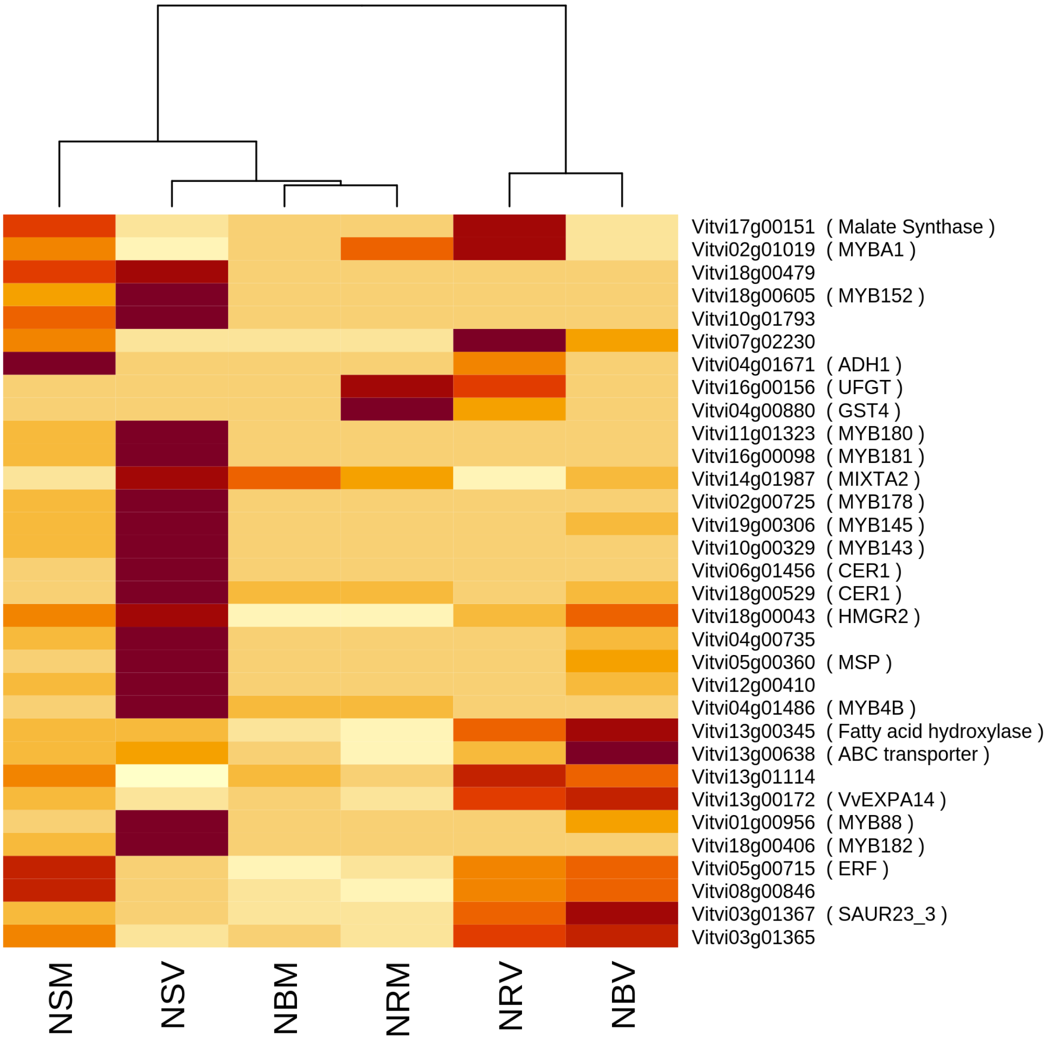

Supplementary Figure S7: Comprehensive heatmap illustrating the dynamic expression patterns (TPM) of key genes discussed throughout the paper, providing a visual representation of their relationship with the studied genotypes.

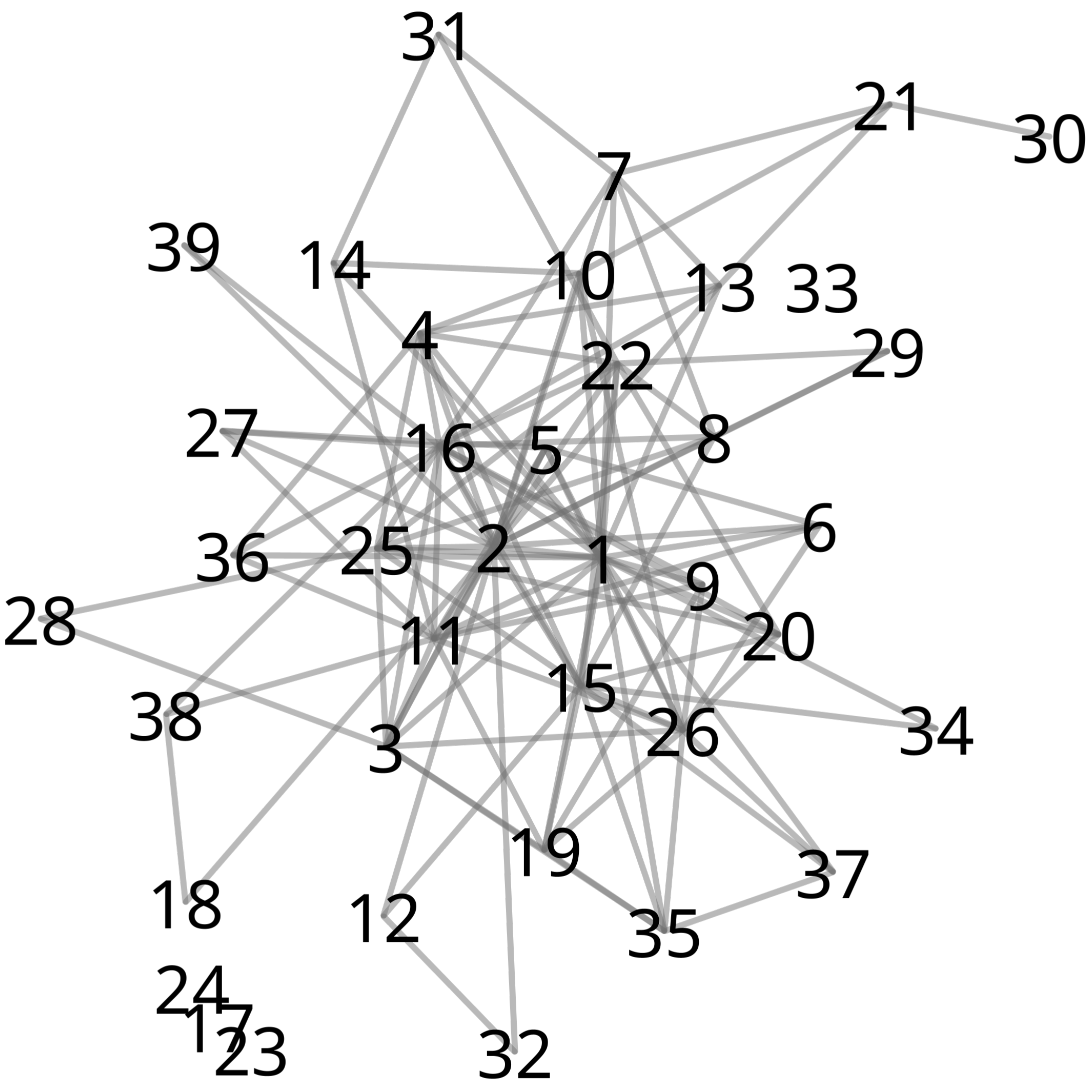

Supplementary Figure S8: WGCNA global co-expression network. Vizualization of the global expression network showcasing modules denoted by their respective module number.

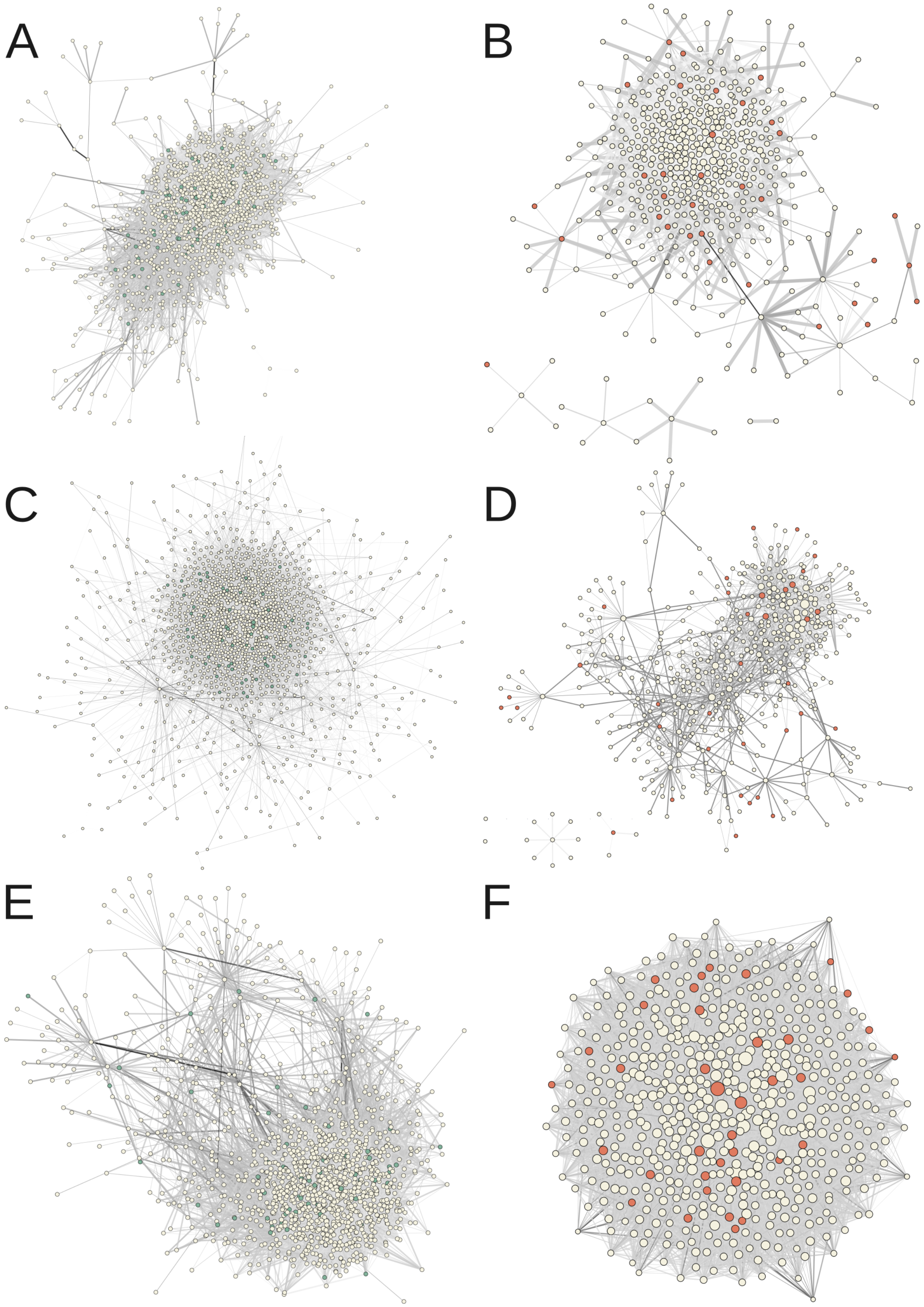

Supplementary Figure S9: Genotype-specific network analysis with the Highest Reciprocal Rank (HRR) method. A-B: Niagara Branca. C-D: Niagara Rosada. E-F: Niagara Steck. The network highlights green modules corresponding to genes from the **SP-AB** intersection group, and orange modules corresponding to genes from the **SN-AB** intersection group.
